# An *Arabidopsis* Tissue-Specific RNAi Method for Studying Genes Essential to Mitosis

**DOI:** 10.1371/journal.pone.0051388

**Published:** 2012-12-07

**Authors:** Brunilís Burgos-Rivera, R. Kelly Dawe

**Affiliations:** 1 Department of Genetics, University of Georgia, Athens, Georgia, United States of America; 2 Department of Plant Biology, University of Georgia, Athens, Georgia, United States of America; Virginia Tech, United States of America

## Abstract

A large fraction of the genes in plants can be considered essential in the sense that when absent the plant fails to develop past the first few cell divisions. The fact that angiosperms pass through a haploid gametophyte stage can make it challenging to propagate such mutants even in the heterozygous condition. Here we describe a tissue-specific RNAi method that allows us to visualize cell division phenotypes in petals, which are large dispensable organs. Portions of the *APETALA* (*AP3*) and *PISTILLATA* (*PI*) promoters confer early petal-specific expression. We show that when either promoter is used to drive the expression of a beta-glucuronidase (GUS) RNAi transgene in plants uniformly expressing GUS, GUS expression is knocked down specifically in petals. We further tested the system by targeting the essential kinetochore protein CENPC and two different components of the Spindle Assembly Checkpoint (MAD2 and BUBR1). Plant lines expressing petal-specific RNAi hairpins targeting these genes exhibited an array of petal phenotypes. Cytological analyses of the affected flower buds confirmed that *CENPC* knockdown causes cell cycle arrest but provided no evidence that either *MAD2* or *BUBR1* are required for mitosis (although both genes are required for petal growth by this assay). A key benefit of the petal-specific RNAi method is that the phenotypes are not expressed in the lineages leading to germ cells, and the phenotypes are faithfully transmitted for at least four generations despite their pronounced effects on growth.

## Introduction

There are roughly 25,000 protein-coding genes in *Arabidopsis thaliana*, and approximately 11% are thought to be essential in the sense that the plant cannot survive without them [1]–[6]. This subset of genes is difficult to study by genetic means because the most informative mutants are dead. Several authors have described methods for inducing mutant phenotypes of essential genes under prescribed conditions, for instance by using chemical inducers or heat-inducible promoters. The chemical induction systems are widely used but involve foreign inducers which can themselves cause growth defects and can be difficult to reproduce [7]. Similar arguments can be made against heat-inducible systems, as hundreds of genes are transcriptionally regulated in response to temperature changes in *Arabidopsis* [8]–[10]. Tissue specific gene silencing is another approach that could in principle be used to study the phenotypes of essential genes. Tissue specific RNAi has been used to knock down genes that control particular developmental outcomes, for instance genes that affect hypodermis or sperm development in animals [11], [12], or genes that control flower morphogenesis [13].

The genes involved in controlling cell division provide a case study of the challenges involved in studying essential genes. Centromeric Protein C (CENPC) is an essential kinetochore foundation component required for accurate cell division in yeast and mammals [14]–[21]. The maize and *Arabidopsis* homologues of CENPC display characteristics and localization patterns that mirror what has been established in other species [22]–[24] and it is presumed that *CENPC* mutants would be non-viable, though this has never been tested. Another interesting but understudied class of genes function in the Spindle Assembly Checkpoint (SAC) pathway, which is typified by the genes *MAD2* (Mitotic-Arrest Deficient) and *BUBR1* and *BUB3* (Budding Uninhibited by Benzimidazole) [25], [26]. In animals, SAC proteins are known to interact with kinetochores to serve a critical surveillance function that delays anaphase until the chromosomes have aligned at metaphase. Mutations in SAC components result in mis-segregation, aneuploidy and cancer [25], [26]. In plants, MAD2 and BUBR1 proteins are localized to kinetochores when mitosis is impaired [27], [28], but may also localize to spindles (unlike in animals) [29]. It is not known if plant SAC proteins have similar roles in regulating anaphase, however, *mad2* mutants are viable, with minor root phenotypes and no other gross abnormalities [30].

One of the more appealing features of flowering plants is their petals, which are for the most part entirely dispensable. They are large, visible organs, not required for growth or reproduction, and the genes that encode for flower identity have been extensively studied. *APETALA* (*AP3*) and *PISTILLATA* (*PI*) are floral homeotic genes required for petal and stamen development in *Arabidopsis* [31]–[33]. Portions of the *AP3* or *PI* promoters (288 bp or 300 bp upstream of the start codons, respectively) confer petal-specific expression during the cell division phase of growth [32], [33]. In this study we show that these promoter regions can provide the basis for effective petal-specific RNAi (psRNAi) vectors that can be used to knockdown the expression of genes involved in mitosis. The data demonstrate that *CENPC*, *BUBR1* and *MAD2* are required for cell division.

## Results

### Portions of the APETALA3 and PISTILLATA promoters are expressed exclusively in petals

**Figure 1:**
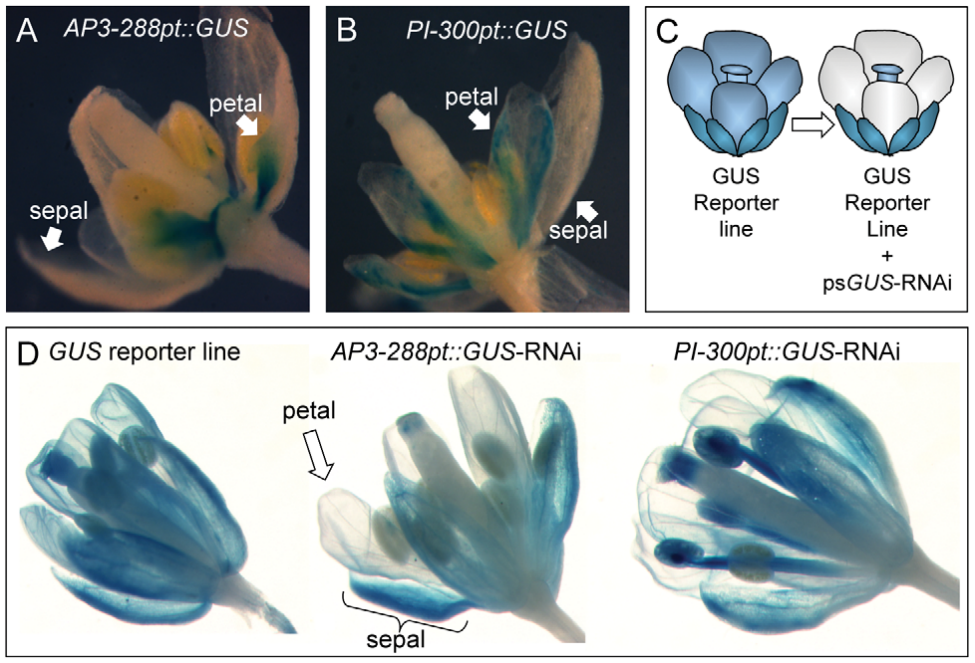
Setting up the petal-specific RNAi system. (**A, B**) Confirmation that the *AP3-288* and *PI-327* promoters fused to GUS show petal-specific *GUS* expression. (**C**) Cartoon showing the expectations when a GUS hairpin construct is driven by the *APETALA* or *PISTILLATA* promoters in a line that uniformly expresses GUS (*ADF9::GUS*). Only the petals should fail to stain with X-Gluc. (**D**) Proof of concept experiment. Both petal specific promoters were used to drive *psGUS-RNAi* in the GUS reporter line background. As shown, the petals were colorless while all other tissues stained blue with X-Gluc.

Previous studies have reported that discrete portions of the *AP3* and *PI* promoters can drive petal expression [32], [33]. A 288 bp *AP3* promoter fragment was reported to be petal specific, whereas a 300 bp *PI* promoter fragment was reported to drive expression in both petals and stamens. In order to confirm these results we fused the sequences to the GUS coding sequence for expression analyses (although we used a slightly longer 327 bp segment of the *PI* promoter). Both *AP3-288pt::GUS* and *PI-327pt::GUS* are expressed in young and developing petals, with little to no expression in mature or senescing petals (Figure 1A,B). There was a small yet noticeable amount of GUS staining in the stamens of lines carrying the *AP3-288pt::GUS* construct, but not in lines carrying the *PI-327pt::GUS* construct, which appeared to be petal specific.

### Petal-specific transgene silencing in Arabidopsis

In order to test the specificity and efficiency of the *AP3* or *PI* promoters in driving the expression of RNAi, we developed RNAi vectors targeting 300 bp of the *GUS* coding sequence. The *AP3-288pt::GUS-RNAi* or *PI-327pt::GUS-RNAi* constructs were introduced into a line constitutively expressing GUS (*ADF9::GUS*,[34]). The ps*GUS*-RNAi transgenes are BASTA resistant, while the *GUS* reporter line is Hygromycin resistant. Plants that showed resistance to both chemicals were screened with X-gluc for *GUS* expression (Figure 1C). We observed that both RNAi constructs removed color from the petals (Figure 1D), with the *AP3-288pt::GUS-RNAi* lines also showing a slight reduction in stamen staining. In the *PI-327pt::GUS-RNAi* line, the petals were white on otherwise blue flowers in 18 of 21 plants that were Hygromycin and BASTA resistant. The numbers for the *AP3-288pt::GUS-RNAi* construct were similar, with 17 of 18 plants containing both transgenes having white petals. The remaining plants showed weak petal staining, presumably reflecting the fact that RNAi is inherently variable. Petal shape and maturation appeared to be completely normal. These experiments were repeated for two generations with similar outcomes (data not shown).

### Petal-specific down-regulation of CENPC

**Figure 2:**
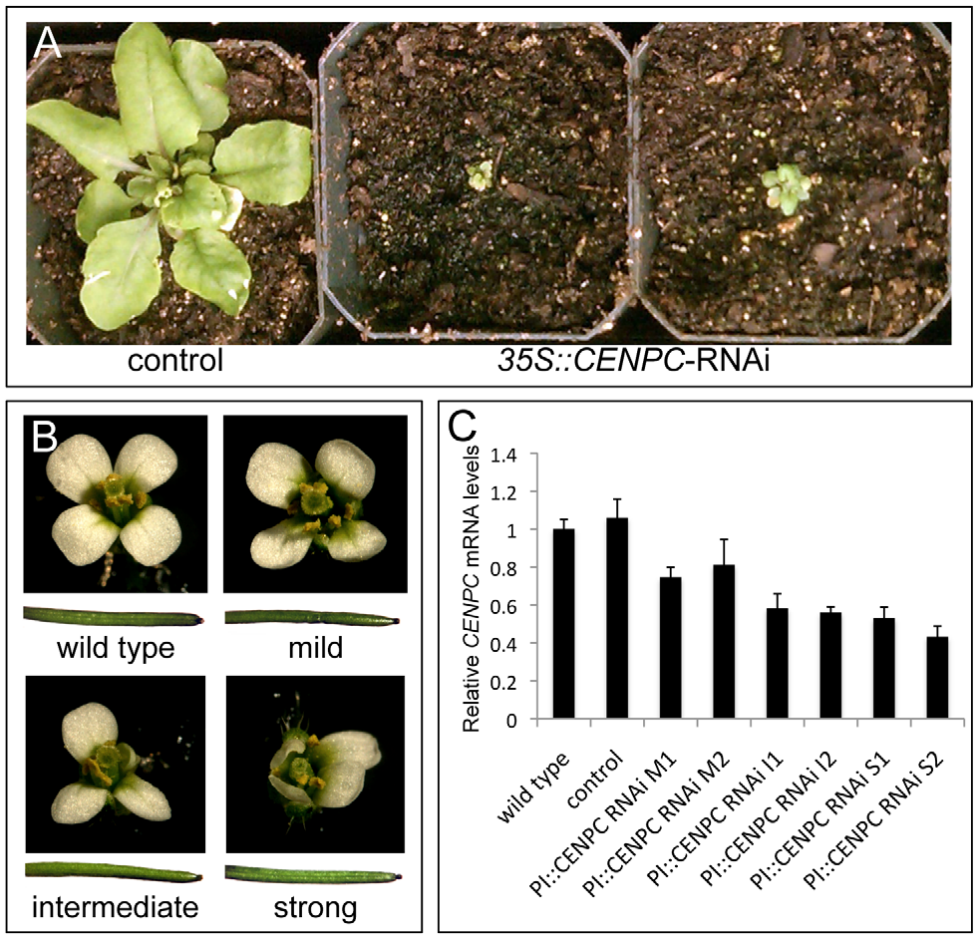
*CENPC* RNAi phenotypes. (**A**) Whole plant CENPC RNAi phenotypes. Plants carrying the *35S::CENPC*-RNAi transgene were arrested at the seedling stage when compared to the control lines. These pictures were taken 28 days after germination. (**B**) Petal-specific *CENPC* RNAi phenotypes. Lines carrying the *PI::CENPC*-RNAi transgene were divided into three categories. Mild lines have four petals and are relatively healthy when compared to wild type. Intermediate and Strong lines are often missing petals or have one or more undeveloped petals. The reproductive organs in all lines were intact and produced normal siliques (shown below each flower). (**C**) mRNA levels in immature flowers of psRNAi lines. Six independent *PI::CENPC*-RNAi T2 lines were assayed (Mild #1, Mild #2, Intermediate #1, Intermediate #2, Strong #1, Strong #2). Wild type and empty vector lines were used as controls. Bars represent the standard error from three technical replicates of each cDNA sample. There was significantly less *CENPC* mRNA in psRNAi lines than in wild type controls (p<0.05).

In fungi and animals, *CENPC* is essential for cell division and subsequent growth. To test whether this is also true in Arabidopsis, we carried out a whole-plant RNAi experiment targeting the first 450 bp of the *AtCENPC* coding sequence using the constitutive CaMV 35S promoter. The transgenic plants stopped growth and died at the young seedling stage (Figure 2A). These results illustrate the weakness of using whole-plant knockdowns (or knockouts) for essential genes: the plants are not available for study. In a successful tissue-specific RNAi system, most of the cells in organism are normal and unaffected, while a dispensable organ such as the petal is subjected to the detrimental effects of the knockdown.

Accordingly, we targeted the first 450 bp of the *AtCENPC* coding sequence for psRNAi using *PI-32*7 as the driving promoter. We recovered 22 independent transformation events. Of these, five exhibited a strong petal phenotype in the form of small, stunted petals. Six other lines showed an intermediate petal phenotype, while the rest had a mild phenotype almost indistinguishable from wild type flowers (Figure 2B). No other leaf or flower organ was affected by the psRNAi transgene: sepals, pistil, carpels, ovules, stamens, anthers, and pollen all appeared normal. The plants produced normal siliques with a full seed set (Figure 2B). To confirm that *CENPC* was down-regulated, we analyzed young flower buds by quantitative RT-PCR. The data showed a statistically significant down-regulation of *CENPC* in lines that had stunted petals relative to wild type (Figure 2C). The ps*CENPC*-RNAi phenotype was heritable and stable for up to three generations (data not shown).

**Figure 3:**
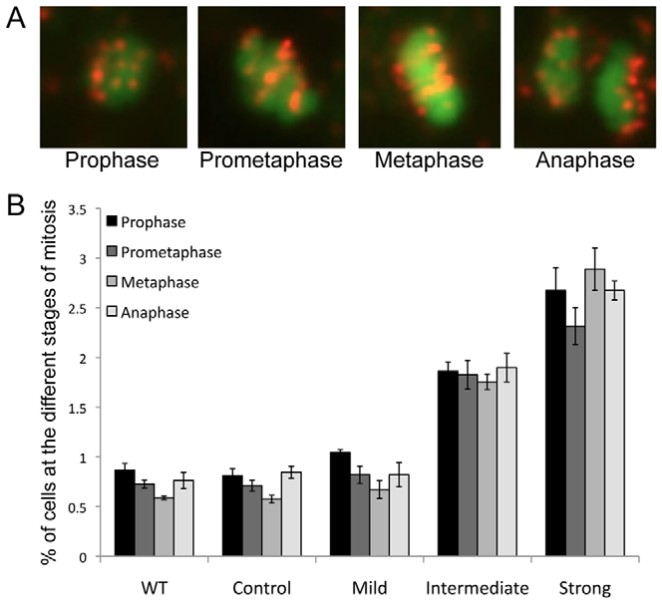
Effects of ps*CENPC*-RNAi on the frequency of cells in mitosis. (**A**) The stages of mitosis scored. Centromeres were identified with a Cen180 FISH probe (red) and DNA was stained with DAPI (green). (**B**) The frequency of mitotic cells in immature flower buds. Bars represent the standard error among biological replicates. There were significant increases (p<0.002) in the percentage of cells at all stages of mitosis in the Intermediate and Strong lines when compared to the Mild, empty vector Control, and wild type (WT) lines.

A loss of CENPC would be expected to cause defects in cell division. To assay mitotic phenotypes we processed immature flower buds that contained developing petals (and multiple other tissues) for fluorescent *in situ* hybridization (FISH). Fixed cells were hybridized with a centromeric DNA probe (Cen180) that labels each of the five *Arabidopsis* centromeres [35], [36]. We scored four stages of mitosis: prophase, where the chromosomes are condensed but unorganized; prometaphase, where the chromosomes are beginning to align; metaphase, where the chromosomes have fully aligned; and anaphase where the chromosomes have segregated to poles (Figure 3A). We found that in our intermediate and strong ps*CENPC*-RNAi lines, there was a significant increase (p<0.002) in the percentage of cells found at prophase, prometaphase, metaphase and anaphase; that is, significantly more cells undergoing the process of chromosome segregation (Figure 3B). Strong lines showed the greatest increase of cells found at each stage, which also correlated with the degree of *CENPC* down-regulation.

### Petal-specific down-regulation of MAD2 and BUBR1

**Figure 4:**
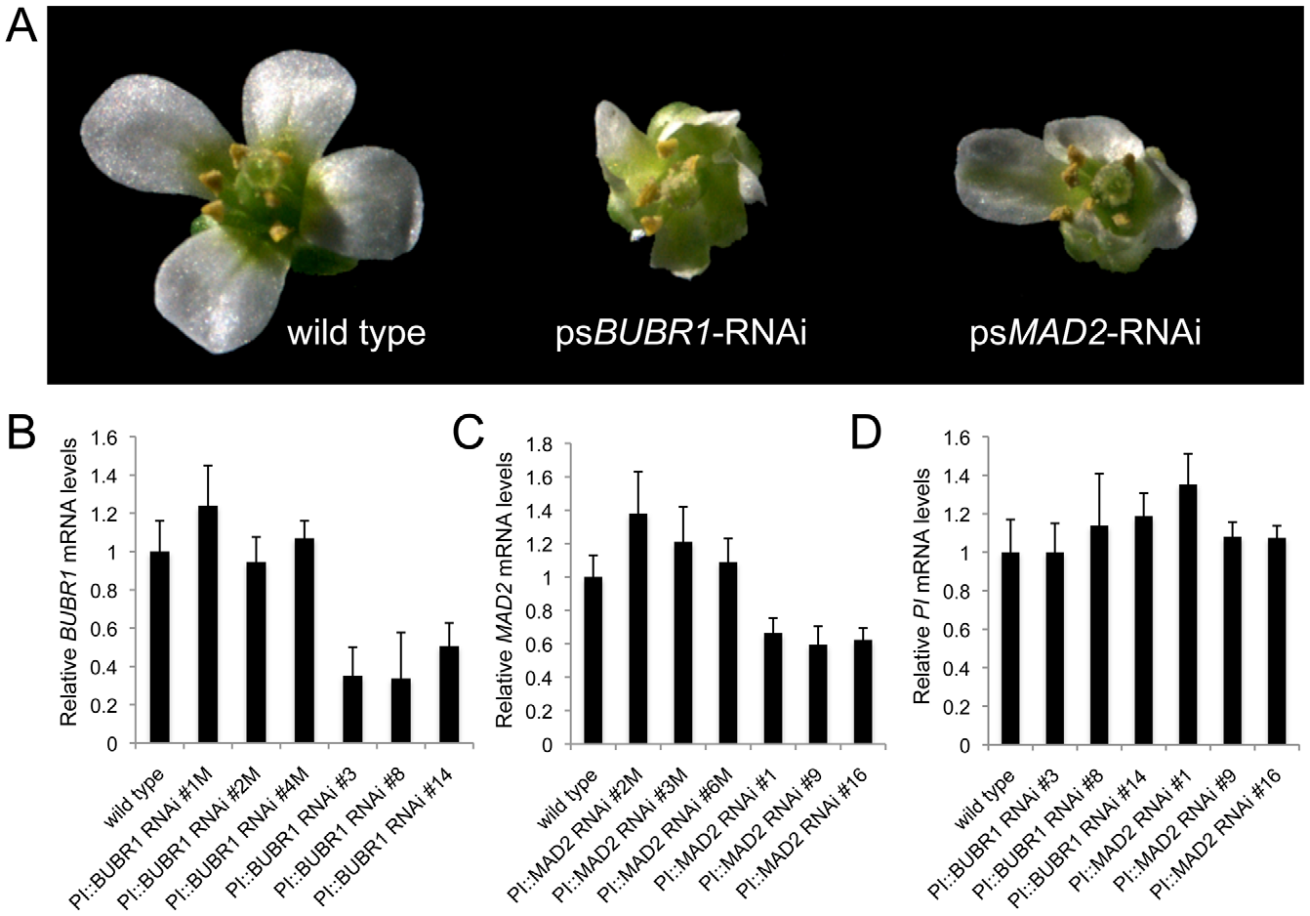
Effects of targeting SAC genes by petal-specific RNAi. (**A**) Lines carrying petal-specific RNAi transgenes targeting *BUBR1* or *MAD2* show petal size and morphology defects when compared to wild type. (**B, C**) The psRNAi constructs significantly reduced mRNA from the targeted genes in immature flower tissue (p<0.05). The first three lines in each panel, noted with an “M” suffix, are those that did not have noticeable petal phenotype. The last three lines in each panel showed petal stunting. Bars represent the standard error from three technical replicates of each cDNA sample. (**D**) Expression of the *PISTILLATA* gene was not affected in lines with stunted petals.

The *PI-327* promoter was also used to drive the expression of hairpin constructs that targeted 344 bp of the *MAD2* or 399 bp of the *BUBR1* coding sequences. We recovered 13 and 11 independent transformation events for ps*BUBR1*-RNAi and ps*MAD2*-RNAi, correspondingly. Four of the ps*BUBR1*-RNAi lines exhibited a petal phenotype in the form of small, stunted petals, similar to the strong petal phenotype observed in ps*CENPC*-RNAi lines (Figure 4A). Likewise, we identified three ps*MAD2*-RNAi lines with a pronounced petal phenotype. The remaining lines had a mild phenotype almost indistinguishable from wild type flowers. The petal phenotypes were observed for up to three generations tested. As for *CENPC*, reductions in the expression of both *MAD2* and *BUBR1* were detected using quantitative RT-PCR (Figure 4B,C). The ps*BUBR1*-RNAi and ps*MAD2*-RNAi lines that exhibited both the petal phenotype and down-regulation of the target mRNA were assayed by FISH. We observed no significant differences between wild type and the *MAD2* or *BUBR1* psRNAi lines in terms of the number of cells in mitosis, or the accuracy of mitosis (no lagging chromosomes or mininuclei were observed).

The petal phenotypes observed in the ps*BUBR1*-RNAi and ps*MAD2*-RNAi lines came as a surprise as there were no flower abnormalities reported in a study of a *MAD2* mutant line [30]. One possible explanation could be that the endogenous PISTILLATA gene is being silenced since the hairpin constructs included the 5′ UTR of *PI*. According to published results, there are two transcriptional start sites within this region, which should produce 83 or 51 nucleotides of mRNA homologous to the *PI* transcript [33]. This sequence is not a part of the predicted hairpin, however there could be indirect transitive RNAi effects on this region. The UTR sequence could also in principle induce RNA-dependent DNA methylation (RdDM) at the endogenous *PI* locus [37]. In order to rule out these affects, we used qPCR to measure *PI* expression levels. No reductions in *PISTILLATA* mRNA were detected in the ps*BUBR1*-RNAi or ps*MAD2*-RNAi lines showing a petal phenotype (Figure 4D).

## Conclusions

We have developed a tissue-specific RNAi system in *Arabidopsis* that can be used to silence both transgenes and endogenous genes, including those that are essential. We successfully targeted *CENPC*, *BUBR1*, and *MAD2* in petals and were able to attribute the petal phenotypes to the down-regulation of the genes. A major strength of this method is that petals are produced late in development from regions of the floral meristem that are separate from the lineages that produce the germ tissues (anthers or carpels) [38]. As such, psRNAi does not affect either plant growth or reproduction, and the phenotypes are reliable and strongly heritable for several generations. Either RNAi or synthetic microRNAs [37], [39] can presumably be used with similar outcomes and any essential gene can be targeted as long as it is expressed in petals.

The literature from animal cells suggests that inactivation of CENPC causes mitotic delay and chromosome mis-segregation [18], [21]. We assayed young flower buds for defects in cell division but found no apparent errors; instead, there was an increase in all mitotic stages that correlated with the severity of the petal stunting phenotype (Figure 3). These data imply that *CENPC* knockdown causes cell cycle arrest at multiple stages. It is also possible (though less likely) that the petal cells failed to enter mitosis altogether, and the dividing cells we scored were derived from other actively dividing tissues. In either case, the data are consistent with a role for CENPC in mitosis. It may be possible to develop more comprehensive ways of measuring petal cell division in the future, for instance by generating double transgenic lines combining ps*CENPC*-RNAi with fluorescent tags that identify the petal cells in the complex flower bud tissues. Such an approach could allow us to directly observe chromosome movement in petal cells (or fixed specimens) and further investigate the effects of *CENPC* knockown.

The literature for spindle checkpoint proteins MAD2 and BUBR1 in plants is primarily limited to immunofluorescence images showing kinetochore localization [27], [28] and descriptions of their behavior in live cells [29]. More recently, a study of MAD1 and MAD2 reported that a *mad2* mutant displayed no gross phenotypic alterations except for a minor defect in root growth [30]. These published results appear to differ qualitatively from our data, which suggest that MAD2 is required to complete normal petal growth (Figure 4). However the results are consistent in suggesting that MAD2 is required to complete cell division on cue, and that in its absence, growth is slowed but not abolished [30]. No mutants or knockdowns of *BUBR1* have been published, but our data suggest that its function is similar to that of *MAD2*.

The apparent discrepancy between published work on a *mad2* mutant and our RNAi data raises the concern that psRNAi may have off-target affects on other genes. We can imagine that knockdowns in any number of genes could have the affect of reducing petal cell proliferation. Although we did not test other genes that may be involved in cell proliferation, it seems clear that mRNA levels from the targeted genes *MAD2* and *BUBR1* are reduced (Figure 4). It is also unlikely that the psRNAi system itself causes petal defects, since the GUS psRNAi experiments did not produce petal morphology defects (Figure 1), and the levels of *PISTILLATA* mRNA are were not affected (Fig. 3). Off target effects can be reduced by more carefully engineering the RNAi constructs, for instance by designing artificial microRNAS [37].

The *MAD2* and *BUBR1* phenotypes are not likely to be caused by cell cycle arrest, as there were no differences in the percentage of cells found in different mitotic stages. Nor did we detect evidence of chromosome loss (such as mininuclei) that might suggest there is a higher mitotic error rate. Although SAC proteins are almost certainly involved in regulating microtubule attachment in plants [27]–[29], they may not be involved in relaying a “wait anaphase” signal to the cell cycle regulatory network. It has been demonstrated that major failures in chromosome alignment during maize meiosis do not significantly delay anaphase progression [40]. It is possible that MAD2 and BUBR1 have important functions in completing cell division and readying the cells for additional growth, perhaps by facilitating the formation of new cell walls in the phragmoplast zone [29].

Previous studies have demonstrated that RNAi is systemic in plants, as small RNAs can move from cell-to-cell or from tissue-to-tissue [41]–[43]. However, small RNAs from hairpin constructs generally move only a few cells from the source, and do not spread by the vasculature. If small RNAs from psRNAi constructs are moving away from petal primordial cells the effects must be minimal, since there were no discernable effects on the surrounding flower tissues, including the reproductive organs (Figures 2B and 4A). The limited movement of the silencing signals may be partially attributable to the fact that petals are one of the last tissues to develop in flowers [38].

## Materials and Methods

### Plant strains and growth conditions

*Arabidopsis* of the Columbia (Col-0) ecotype were grown on soil or agar in growth chambers at 22°C under fluorescent light for 16 h. Transformations were performed with *Agrobacterium tumefaciens* strain C58C1 using the floral dip method [44]. Transformants were selected by plating seeds on half-strength Murashige and Skoog (MS) media [45] containing 50 mg DL-phosphinothricin (GOLD Biotechnology Inc.).

### Petal-specific promoter vectors

The petal-specific domain of the *AP3* or *PI* promoter was amplified with a forward primer (AP3-S: 5′-TACGTCGAATTCAGTGTCTTGTAATTATACAA-3′; PI-S1: 5′-TACGTCGAATTCTTATTACGTTACTTCAAGTT-3′) and a reverse primer (AP3-A: 5′-TAGCTGCCATGGATTCTTCTCTCTTTGTTTAA-3′; PI-A: 5′-TAGCTGCCATGGCTTTCTCTCTCTATCTCTTT-3′) from wild-type Col-0 *Arabidopsis* genomic DNA. The amplified product was cloned into a TOPO TA Cloning® vector (Invitrogen, CA, USA) and sequenced. Cloned plasmid DNA without sequence errors was digested with *EcoRI* and *NcoI* and the petal-specific promoter fragment inserted into the same restriction sites of the *pFGC5941* plasmid (GenBank Accession No. AY310901; Arabidopsis Biological Resource Center stock number CD3-447) to replace the CaMV 35S promoter and generate the *AP3-288pt-pFGC5941* or *PI-327pt-pFGC5941* vectors for petal specific gene silencing. The original plasmid *pFGC5941* is a specialized RNAi vector that accepts the same sequence twice in opposing orientations depending on restriction sites: *AscI/SwaI* inserts a forward orientation, while *BamHI/XbaI* inserts a reverse orientation, such that the two reverse sequences are separated by a CHSAi spacer.

### AP3-288pt::GUS and PI-327pt::GUS plants

The *GUS* reporter gene was amplified with a forward (GUS-S2: 5′-TACGCTCCATTGATGTTAACGTCCTGTAGAA-3′) and reverse (GUS-A2: 5′-TAGCTGTTAATTAATCATTGTTTGCCTCCCTG-3′) and cloned it into a TOPO TA Cloning® vector (Invitrogen, CA, USA) for sequencing. Cloned plasmid DNA without sequence errors was digested with *NcoI* and *PacI* and the full length GUS fragment was inserted in frame into the same restriction sites of the modified *AP3-288pt-pFGC5941* or *PI-327pt-pFGC5941* binary vectors (described above), replacing the CHSAi spacer. Transgenic plants were selected by antibiotic resistance and screened with X-gluc for *GUS* expression [46].

### AP3-288pt::GUS-RNAi, PI-327pt::GUS-RNAi plants

The first 300 bp of the *GUS* coding sequence were amplified using forward (GUS-S: 5′-TACGCTTCTAGAGGCGCGCCCACCGTGGTGACGCATGTCG-3′) and reverse (GUS-A: 5′-TAGCTGGGATCCATTTAAATCAGCAGTTTCATCAATCACCAC-3′) primers and cloned into a TOPO TA Cloning® vector. Cloned plasmid DNA without sequence errors was digested with *AscI/SwaI* or *BamHI/XbaI* to generate two complimentary RNAi fragments. The fragments were subcloned into the same restriction sites of the *AP3-288pt-pFGC5941* or *PI-327pt–pFGC5941* binary vectors (described above).

### PI-327pt::CENPC-RNAi, PI-327pt::MAD2-RNAi, and PI-327pt::BUB1-RNAi, and empty vector control plants

The *CENPC*, *MAD2*, and *BUBR1* RNAi fragments were amplified from wild type Col-0 flower cDNA library (Invitrogen, Carlsband, CA). We amplified the first 450 bp of *CENPC*, 344 bp of *MAD2* and 399 bp of *BUBR1* coding sequences using forward (CENPC-RNAiF: 5′-TACGTCTCTAGAGGCGCGCCATGGCTGATGTGAGCCGGAGTTCAAGTTTATATA-‘3; MAD2-RNAiF: 5′-TACGTCTCTAGAGGCGCGCCATGGCGTCCAAAACAGCGGCTGCTAAAGATAT-‘3; BUB1R-RNAiF: 5′-TACGTCTCTAGAGGCGCGCCATGGCAGCCGAAACGAA-‘3) and reverse (CENPC-RNAiR: 5′-ATCGACGGATCCATTTAAATTATATCTATTACACTGGAGCCAGTCTGTTTCTGCC-‘3; MAD2-RNAiR: 5′-ATCGACGGATCCATTTAAATCCTTTGTCAACAACTTCATTATCAGTCT-‘3; BUB1R-RNAiR: 5′-ATCGACGGATCCATTTAAATCAACCAGACTTTAAGATAACGAAGATCATCC-‘3) primers. The RNAi fragments were cloned into a TOPO TA Cloning® vector (Invitrogen, CA, USA) for sequencing. Cloned plasmid DNA without sequence errors was digested with *AscI/SwaI* or *BamHI/XbaI* to generate complimentary sequences and cloned into *PI-327pt-pFGC5941* as described above. The empty *PI-327pt-pFGC5941* vector was also transformed into *Arabidopsis* in order to generate control lines. Transgenic plants were selected by antibiotic resistance and by the stunted petal phenotype.

### GUS histochemical analysis

GUS histochemical analyses were carried out on lines expressing *AP3-288pt::GUS* and *PI-327pt::GUS*. We assayed a total of twelve independent T2 lines per construct. Plants at various stages including emerging seedling, 7–10-day-old whole seedling, 20-day-old whole plant, and maturing buds and flowers were assayed after four and 16 hours in a GUS staining solution [46].

### Quantitative Real Time PCR analyses

RNA was isolated from flower buds using the Spectrum™ Plant Total RNA Kit (Sigma-Aldrich, St. Louis, MO USA). Total RNA (1 µg) from each sample was transcribed into cDNA with the Super Script III kit (Invitrogen, Carlsbad, CA, USA) following the manufacturer's instructions except that incubations were performed for 30 min at 55°C using an oligo (dT) primer. Aliquots of the cDNA were used as template for the qRT-PCR analyses in triplicate reactions for each of the biological replicates on an Applied Biosystems 7500 Real Time PCR Instrument. Real time PCR reactions consisted of SYBR GREEN PCR Master Mix (Applied Biosystems, Foster City, CA, USA), 0.4 µM of each primer, and 1∶25 dilution of cDNA in a 20 µl reaction volume. Expression of the ubiquitin gene UBQ10 was used as the internal control. Primers were as follows:

CENPC (CENPCqPCR-F4: 5′- GTTTATATACCGAGGAGGATCCCCTTCAAGC -3′; CENPCqPCR-R4: 5′- GGCATAGATTGAAGGAGAGTATGAGTCTGCTG -3′); MAD2 (MAD2qPCR-F1: 5′-AAAACAGCGGCTGCTAAAGA-3′; MAD2qPCR-R1: 5′-TTCGCAGCGTAACAGAAGAA-3′); BUB1 (BUB1RqPCR-F1 5′-ATTCGAAGCAGGAGACTGGA-3′; BUB1RqPCR-R1 5′-ACCAACATTGCGACCTCTCT-3′); PISTILLATA (PI-qPCR_F1: 5′-ACAACTGGAGCTCAGGCATT-3′;

PI-qPCR_R1: 5′-GACTTTGTCGAGGCCATGTT-3′) and UBQ10 (UBQ-RTS: 5′-AGAAGTTCAATGTTTCGTTTCATGTAA-3′, UBQ-RTA: 5′-GAACGGAAACATAGTAGAACACTTATTCA-3′).

### Fluorescence in situ hybridization and cytological counts

Immature flower buds were harvested from one wild type and one empty vector lineage, two independent Mild, Intermediate and Strong CENPC psRNAi lines, and three independent *BUBR1* and *MAD2* psRNAi lines showing the petal phenotype. For each line, the immature buds from at least three different plants were pooled. Therefore each construct is represented by biological replicates (lines representing independent transformation events) and technical replicates (pooled tissues from multiple plants). The tissue was fixed and digested for FISH analyses as described in the *Arabidopsis* Protocols book [47]. Centromeres were identified using a cy3-labeled Cen180 probe consisting of three synthesized oligonucleotides (cen180_oligo2: 5′- Cy3/GGTGTAGCCAAAGTCCRTATGAGTCTTTGK-3′; cen180_oligo5: 5′- Cy3/TCTTATACTCAATCATACACATGACATCW-3′; cen180_oligo6: 5′- Cy3/AGTCATATTYGACTCCAAAACACTAACC-3′). DNA was stained using ProLong® Gold antifade reagent with DAPI (Life Technologies). Cells were viewed using a Zeiss Axio Imager, and images were collected using Slidebook 4.0 software (Intelligent Imaging Innovations). On average, we counted 1000 cells from each line and classified cells by mitotic stage (prophase, prometaphase, metaphase, and anaphase). The data from biological replicates were averaged and plotted. Error bars represent standard error. A *t*-test (2 tail distribution, equal variance assumed) was used to compare each treatment to the wild type control.
